# Efficacy and Safety of Transarterial Radioembolization in Elderly Patients with Hepatocellular Carcinoma: A Single-Center Experience

**DOI:** 10.5152/tjg.2024.23155

**Published:** 2024-03-01

**Authors:** Ümit Karaoğullarından, Yüksel Gümürdülü, Oğuz Üsküdar, Emre Odabaş, Hasan Selim Güler, Cansu Geçkalan, Burcu Arslan Benli, Anıl Delik, Asena Ayça Özdemir, Sedef Kuran

**Affiliations:** 1 Department of Gastroenterology, Çukurova University, Adana, Turkey; 2 Department of Medical Education, Mersin University, Mersin, Turkey

**Keywords:** Elderly, hepatocellular carcinoma, safety, survival, transarterial radioembolization

## Abstract

**Background/Aims:**

Hepatocellular carcinoma is a major cause of mortality and morbidity in both cirrhotic and non-cirrhotic patients, and most patients are suitable for locoregional and/or systemic therapy at the time of diagnosis. In this study, we aimed to determine the efficacy and safety of transarterial radioembolization in elderly patients.

**Materials and Methods:**

Patients diagnosed with hepatocellular carcinoma between 2013 and 2022 were screened retrospectively. The patients were divided into 2 groups: the elderly (age ≥70 years) and the young (age <70 years). Transarterial radioembolization response was evaluated according to the Response Evaluation Criteria in Solid Tumors.

**Results:**

Ninety patients were included in the young group, and 56 patients were in the elderly group**. **It was observed that male dominance was less in the elderly group (*P* > .05). Hepatitis B was the most common cause in both groups. There were no significant differences between groups with regard to morphological features of tumors [tumor focality (single; 62.2% and 60.7%, respectively) and maximal tumor diameter (6.9 and 6.55 cm, respectively)], transarterial radioembolization responses (51.1% and 39.3%, respectively), survival (9 and 8.5 months), and both early and late side effects (*P* > .05). Age was not found to be an effective factor in transarterial radioembolization response (*P* > .05).

**Conclusion:**

No differences in the safety and efficacy of transarterial radioembolization were observed between the groups. In addition, it was observed that age was not a predictive factor for adverse events. In elderly patients in the frail group, it should be considered that age alone should not be seen as a limitation in the transarterial radioembolization decision.

**Main Points**

- Transarterial radioembolization (TARE) procedure is safe and effective in older patients.
- Age is not a risk factor for side effects in the TARE procedure.
- When a TARE decision is made, age alone should not be a limitation.
- It is necessary to act more boldly when making TARE decisions for elderly patients.

## Introduction

Hepatocellular carcinoma (HCC) is one of the most common causes of cancer-related deaths.^1^ Advanced age is an important factor in the development of HCC. Both the incidence of HCC and the risk of HCC increase with age.^1,2^ As life expectancy increases, the choice of treatment in older patients has become more important. Both their performance and comorbid diseases create serious limitations. In addition to these limitations, knowing the safety and effectiveness of treatment modalities in older patients plays a vital role.

Hepatocellular carcinoma treatment selection is made according to current guidelines and with a multidisciplinary approach.^3^ Patients are mostly evaluated according to the Barcelona Clinic of Liver Cancer (BCLC) Classification. According to the BCLC Classification, 70% of patients are in stages BCLC B or BCLC C at the time of diagnosis and are suitable for locoregional therapy and/or systemic therapy.^4^

Transarterial radioembolization (also known as TARE, selective internal radiation therapy [SIRT]) has been shown in many studies to be an effective treatment for patients with moderate and advanced HCC.^5-9^ When we searched the literature, there were very few studies on its efficacy and safety in older patients.

In this study, we aimed to determine the efficacy and safety of TARE in elderly patients.

## Materials and Methods

Patients diagnosed with HCC between 2013 and 2022 were screened. The inclusion criteria were as follows: age >18 years, radiologic and/or histologic diagnosis of HCC, having undergone TARE at least once, preprocedure triphasic computed tomography (CT) and/or dynamic magnetic resonance imaging (MRI), and triphasic CT and/or dynamic MRI after the procedure.

The definition of elderly patients is another important issue. Many cutoffs are used in the 65-80 years age range. Both the International Society of Geriatric Oncology and the scientific literature currently use the age limit of 70 years to define a patient as elderly.^10^ Therefore, we divided the patients into 2 groups, the elderly and the young group (age ≥70 years and <70 years, respectively).

Ineligibility criteria for transarterial chemoembolization (TACE) were portal vein thrombosis (major trunk) and increased tumor burden. For TARE treatment, all patients were identified according to current international guidelines.^3,11^

Demographic data, comorbid diseases, liver reserves [according to the Child–Pugh score (CPS)], cirrhotic and noncirrhotic backgrounds, performance status, post-TARE adverse effects, post-TARE performance shifts, and post-TARE CPS shifts were recorded. All information was recorded from the hospital’s electronic data system.

Evaluation after TARE was carried out at 8-12 weeks from the procedure as recommended.^3^

All adverse effects after TARE up to response assessment were recorded.

Serious adverse effects after TARE such as new-onset ascites and/or encephalopathy after TARE and/or Child–Pugh C score and TARE-related death were determined.

The TARE procedure plan was evaluated and decided upon by a multidisciplinary committee consisting of a gastroenterologist, interventional radiologist, oncologist, and hepatobiliary surgeon. The evaluation was based on the BCLC Classification with patient-specific approaches.

According to the Eastern Cooperative Oncology Group (ECOG) scale, physiological reserve and functional status were evaluated in cancer patients.^12^

Overall survival was calculated from the time of HCC diagnosis (months).

Ethics committee approval from Çukurova University (125-79/2022) was obtained for the study. The study complies with the revised Declaration of Helsinki. Written informed consent was obtained from the patients who agreed to take part in the study

### Transarterial Radioembolization Procedure

Angiography was performed in all patients before undergoing TARE. To prevent the spread of Y-90 microspheres to nontarget areas, the vascular structure was examined in detail. Technetium-99m macroaggregated albumin (99mTc-MAA) was administered, and then hepatic artery perfusion scintigraphy was performed. Transarterial radioembolization was not performed on those with severe lung shunt (>20%), severe tumor burden (>60%), and extrahepatic uptake.

Transarterial radioembolization/SIRT procedures were performed in the standard way with yttrium (Y-90) microspheres. The procedure was performed as subsegmental, segmental, or lobar, depending on the location of the tumor.

Response assessment was performed using dynamic MRI or triphasic CT.

The response was evaluated according to the Response Evaluation Criteria in Solid Tumors (RECIST).^3^

### Statistical Analysis

Continuous variables were evaluated using the Shapiro–Wilk test. Mann-Whitney *U*-test was used for comparisons made according to age groups. Chi-square and Fisher’s precision tests were used in the analysis of categorical data. Univariate logistic regression analysis and multiple logistic regression analysis were used to determine the risk factors affecting adverse events. For statistical analysis, IBM Statistical Package for the Social Sciences version 21.0 program (IBM Inc., Armonk, NY, USA) was used.

## Results

The general characteristics of the patients are given in Table 1. There was significant male dominance in both groups. Male dominance was less in the elderly group (*P* > .05). The Eastern Cooperative Oncology Group scale scores between groups were similar (*P* > .05). Previous treatments were similar between groups (*P* > .05). Diabetes mellitus and hypertension were found significantly more frequently in the elderly group (*P* < .05). Hepatitis B was the most common cause in both groups. There was no significant difference between the groups in terms of morphological features (tumor focality and maximal tumor diameter) of the tumors (*P* > .05). Barcelona Clinic of Liver Cancer Classification distribution was similar between groups (*P* > .05). Transarterial radioembolization targets and responses were similar between groups (*P* > .05). Survival times were similar between groups (*P* > .05).

Transarterial radioembolization adverse effects are given in Table 2. All adverse effects that occurred after TARE and before the radiologic evaluation were evaluated. Adverse event rates were similar in both groups (*P* > .05).

The factors affecting surveillance in older patients are given in Table 3. In older patients, only portal vein thrombosis and unresponsiveness to TARE or the presence of progressive response were found to be effective on the surveillance (P < .05). In the multivariate analysis, only unresponsiveness to TARE was an independent risk factor for survival (P < .05).

The factors affecting TARE response in older patients are given in Table 4. Age was not found to be effective on TARE response (*P* > .05). In the univariate analysis, it was observed that neither the patient’s performance status nor sex, nor morphologic characteristics of the tumor, nor alpha-fetoprotein (AFP) levels, nor presence of PVT, nor cirrhotic background, nor BCLC classification of the tumor was effective on TARE response (*P* > .05).

## Discussion

The world population is aging and the risk of HCC, like many other cancers, increases with age. Older people are also underrepresented in clinical trials. The efficacy and safety of treatments in older patients are unclear.

We found that TARE was as effective and safe in older patients as in younger patients. In older patients, the adverse-effect profile was similar to younger patients. We observed that the only independent risk factor effective in the surveillance of older patients was the response to TARE. We found that age was not an effective factor in the response of TARE in older patients.

According to current guidelines, TACE is the main treatment option for intermediate stage HCC. There are many studies comparing TARE with TACE. In a comprehensive meta-analysis on this subject, patients who underwent 461 TARE and 1096 TACE were compared. Overall survival, tumor response, and safety profile were similar in both locoregional treatments. It was observed that progression-free survival was better in patients who underwent TARE.^13^

In our study, there was significant male dominance and etiological HBV in the elderly and young groups. Male dominance and HBV were slightly less in the elderly group than in the younger group.

In a recent study, in a cohort of 407 patients, the age limit was determined as 70 years in the elderly and young groups, similar to our study. It was observed that there was a similar male dominance in the elderly group, but less dominance when compare to the young group.^14^ In a multicenter retrospective study conducted on 1718 patients, male dominance was observed in both groups, and male dominance was observed to be slightly less in the elderly group (≥70 years). When evaluated as an etiologic factor, HCV was the most common factor in both groups.^15^

In our study, in patients with HCC who underwent TARE, there was no difference in previous treatment modalities between the elderly and young patient groups. In a recent study, in general, treatment modalities were compared in older patients and young patients with HCC, and transplantation was observed significantly more in the younger patient group and supportive care in the older patient group.^14^

In a high-volume multicenter retrospective study, treatments were generally evaluated in older and young patients with HCC. It has been observed that ablative treatments are used more frequently in the elderly, whereas TACE and resection are performed less frequently.^15^ However, the differences may be because our study was conducted only on patients who underwent TARE, not on general patients with HCC.

In our study, in patients with HCC who underwent TARE, older patients and young patients were evaluated in terms of tumor morphology. The groups were similar in terms of maximal tumor diameter (MTD) or tumor focality. Our results differed slightly from the literature. Studies were showing that MTD was higher in the elderly group and there was no difference in tumor focality.^14,15^ However, this difference may be related to our study only evaluating the specific group in which TARE was performed.

In the present study, AFP levels and portal invasion rates were similar between the elderly and young groups. Portal invasion rates were similar between the elderly and young populations in high-volume multicenter studies, both in the literature and in agreement with our study.^14,15^

We observed a shift over time in the literature on the effectiveness of TARE. In a study, 52 patients with intermediate and advanced-stage HCC were followed prospectively, and was stated that TARE was an effective treatment modality.^16^ At the beginning of 2010, TARE was shown in many studies as an effective and well-tolerated treatment in patients with moderate and advanced HCC. TARE has been suggested to be suitable for HCCs of BCLC-C stage disease limited to the liver, and it has been reported to be as effective as sorafenib.^17-19^

In prospective studies conducted in 2020, TARE efficacy was compared with sorafenib in patients with intermediate or advanced HCC outside the TACE limits, and no significant superiority of TARE use over sorafenib was observed.^20,21^ In another prospective study, the combination of sorafenib and sorafenib + TARE was compared, and no significant benefit of adding TARE to treatment was observed.^22^

In the literature, studies on the efficacy and safety of TARE in elderly patients are limited.^15,23-25^

In a multicenter (8 centers in Europe), retrospective study investigating the efficacy and safety of TARE, 325 patients were evaluated. Older (≥70 years) and young (<70 years) patients with the same tumor morphologic features (MTD, focality), AFP, and portal invasion were compared. The effects of TARE on tolerability and survival were evaluated, and results were similar across groups. Likewise, their effects on survival were found to be similar.^23^

In the literature, studies on the efficacy and safety of TARE in older patients were mostly related to liver-dominant metastatic colorectal cancers. In a multicenter (USA, 11 centers), retrospective study, 606 patients with liver-dominant metastatic colorectal cancer were evaluated and there was no difference in efficacy and safety in the elderly (age ≥70 years) and younger (age <70 years) groups.^24^ In another study of 107 patients with liver-dominant metastatic colorectal carcinoma, the safety and efficacy of radioembolization between the elderly and young patients were similar.^25^

There were some limitations of our study. First, our study was retrospective. Due to the approximately 10-year duration of this study period, the HCC diagnosis and treatment procedure may not be consistent in every patient, resulting in bias. Likewise, the development of TARE treatment procedures over the years leads to heterogeneity among patients. The findings of the study should not be attributed to the general population because it was a single-center study. Older patients with uncontrolled and multiple comorbidities may not have been given TARE, which may cause bias.

As a result, we observed no differences in the safety and efficacy of TARE between the elderly and young populations. We also found that age was not a predictive factor for adverse events. It should be considered that age alone should not be seen as a limitation when making the decision for TARE in older patients, who are in the frail group. Older patients should not miss an effective treatment option just because they are old. We should be more courageous when making decisions for TARE in older patients. Prospective multicenter studies are needed to determine the safety and efficacy profiles of TARE in the elderly in line with current developments in current TARE procedures and HCC diagnoses.

## Figures and Tables

**Table 1.** General Characteristics of Patients

	Age <70 years		Age ≥70 years		*P*
	Median [IQR] *n*	Minimum–Maximum/%	Median [IQR] *n*	Minimum–Maximum/%	
Number	90	61.6	56	38.4	3
Age	60 [55-66]	35-69	75 [74-78]	70-84	**<** **.001^a^** 3
Male	3	82.2	3	67.9	**.046^b^** 3
ECOG	3	3	3	3	3
	0	28	31.1	18	32.1	.290^b^3
	1	34	37.8	18	32.1	
	2	24	26.7	20	35.7	
3	4	4.4	0	0.0		
Previous treatment	3	3	3	3	3
	Surgical	8	8.9	2	3.6	.449^b^3
	TACE	16	17.8	10	17.9	
	Ablation	22	24.4	12	21.4	
	Systemic therapy	14	15.6	6	10.7	
Supportive care	30	33.3	26	46.4		
Comorbidities	3	3	3	3	3
DM	20	22.2	6	10.7	.077^b^3
HT	24	26.7	20	35.7	.247^b^3
CAD	12	13.3	8	14.3	.871^b^3
CRF	2	2.2	8	14.3	**.007^c^** 3
Presence of cirrhosis	68	75.6	46	82.1	.349^b^3
Etiology	3	3	3	3	3
HBV	40	44.4	20	35.7	.201^b^3
HCV	24	26.7	14	25.0	
NASH	20	22.2	12	21.4	
Other	6	6.7	10	17.9	
CPC	3	3	3	3	3
A	84	93.3	52	92.9	1.00^c^3
B	6	6.7	4	7.1	
Tumor focality	3	3	3	3	3
Single	56	62.2	34	60.7	.855^b^3
Multiple	34	37.8	22	39.3	
MTD	6.9 [4.75-9.23]	3-16	6.55 [4.08-10]	2.9-17	.994^a^3
Portal vein thrombosis	28	31.1	18	32.1	.896^b^3
Ascites	8	8.9	6	10.7	.716^b^3
Encephalopathy	0	0.0	0	0.0	–
BCLC Classification	3	3	3	3	3
A	0	0.0	0	0.0	.239^b^3
B	44	48.9	26	46.4	
C	42	46.7	30	53.6	
D	4	4.4	0	0.0	
Total bilirubin	0.89 [0.64-1.08]	0.19-3.2	0.78 [0.52-1.19]	0.09-3.49	.723^a^3
Albumin	3.9 [3.38-4.1]	2.3-5	3.7 [3.43-4.1]	2.9-4.8	.358^a^3
INR	1.1 [1.04-1.18]	0.93-1.39	1.05 [1-1.17]	0.87-3.6	**.009** ^**a**^3
AFP	36	40.0	12	21.4	**.020** ^**b**^3
TARE target	3	3	3	3	3
Right	58	64.4	34	60.7	.650^b^3
Left	32	35.6	22	39.3	
TARE	3	3	3	3	3
Total	46	51.1	22	39.3	.335^b^3
Partial	38	42.2	28	50.0	
Progressive	6	6.7	6	10.7	
Survival (months)	9 [4-14]	1-41	8.5 [4-21.25]	1-32	.759^a^3

Values in bold signify significantly higher rate (*P* < .05).

AFP, alpha-fetoprotein; BCLC, Barcelona Clinic of Liver Cancer; CAD, coronary artery disease; CPC, Child–Pugh classification; CRF, Chronic renal failure; DM, diabetes mellitus; ECOG, Eastern Cooperative Oncology Group; HBV, hepatitis B; HCV, hepatitis C; HT, hypertension; INR, international normalized ratio; MTD, maximal tumor diameter; NAFLD, nonalcoholic fatty liver disease; TACE, transarterial chemoembolization; TARE, transarterial radioembolization.

^a^Mann–Whitney *U*-test. ^b^Chi-square test. ^c^Fisher’s exact test.

**Table 2.** TARE Side Effects

	Age <70 years		Age ≥70 years		*P*
	Median [IQR] n	Minimum–Maximum/%	Median [IQR] n	Minimum–Maximum/%	
Nausea vomiting	30	33.3	18	32.1	.882^b^3
Fatigue	34	37.8	20	35.7	.802^b^3
Fever	10	11.1	5	8.9	.784^c^3
Hepatic encephalopathy	2	2.2	2	3.6	.638^c^3
Abdominal pain	24	26.7	12	21.4	.475^b^3
Acute kidney injury	10	11.1	6	10.7	.941^b^3
Radiation gastroduodenal ulcer	4	4.4	2	3.6	1.00^c^3
Radiation cholecystitis	4	4.4	2	3.6	1.00^c^3
Biliary complication	12	13.3	6	10.7	.640^b^3
Ascites	10	11.1	4	7.1	.567^c^3
Death	2	2.2	2	3.6	.638^c^3
Post-TARE albumin	3.6 [3.08-4]	1.8-4.4	3.5 [3.1-3.78]	2.5-4.4	.809^a^3
Post-TARE bilirubin	0.96 [0.74-1.21]	0.17-10.7	0.83 [0.54-1.38]	0.32-3.12	.346^a^3

Values in bold signify significantly higher rate (*P* < .05).

TARE, transarterial radioembolization.

^a^Mann–Whitney *U*-test. ^b^Chi-square test. ^c^Fisher’s exact test.

**Table 3.** Factors Affecting Survival in Elderly Patients

	Univariate				Multivariate			
	HR	95% CI Lower	95% CI Upper	*P*	HR	95% CI Lower	95% CI Upper	*P*
Male	0.751	0.291	1.939	.554	3	3	3	3
ECOG	3	3	3	3	3	3	3	3
0	Ref.	3	3	3	3	3	3	3
1	0.637	0.196	2.067	.453	3	3	3	3
2	0.781	0.248	2.464	.674	3	3	3	3
BCLC (C)	2.613	0.969	7.043	.058	3	3	3	3
AFP (>400)	1.760	0.656	4.724	.262	3	3	3	3
Portal vein thrombosis	3.355	1.222	9.208	**.019**3	1.627	0.496	5.341	.422
Multiple	0.590	0.218	1.595	.298	3	3	3	3
Comorbidities	3	3	3	3	3	3	3	3
DM	1.243	0.282	5.477	.774	3	3	3	3
HT	0.731	0.268	1.993	.541	3	3	3	3
CAD	0.839	0.187	3.776	.820	3	3	3	3
CRF	2.165	0.697	6.727	.182	3	3	3	3
CPC	0.043	0.000	84.694	.416	3	3	3	3
TARE response	3	3	3	3	3	3	3	3
Total	Ref.	3	3	3	3	3	3	3
Partial	2.059	0.618	6.860	.239	1.544	0.420	5.678	.513
** **Progressive	11.515	2.626	50.481	**.001**3	12.549	2.560	61.521	**.002**3
Ascites	0.040	0.000	21.502	.315	3	3	3	3
Cirrhosis	3.374	0.724	15.728	.122	3	3	3	3
Albumin	0.340	0.099	1.167	.086	3	3	3	3

*P: *Cox regression; values in bold signify significantly higher rate (*P* < .05).

AFP, alpha-fetoprotein; BCLC, Barcelona Clinic of Liver Cancer; CAD, coronary artery disease; CPC, Child–Pugh classification; CRF, chronic renal failure; DM, diabetes mellitus; HT, hypertension.

**Table 4.** Factors Affecting TARE Response in Elderly Patients

	Univariate	95% CI Lower	95% CI Upper	*P*
	OR			
Age	0.882	0.746	1.043	.143
Male	0.369	0.116	1.171	.091
ECOG	3	3	3	3
0	Ref.	3	3	3
1	4.375	1.027	18.629	**.046**3
2	2.333	0.560	9.717	.244
BCLC (C)	0.583	0.198	1.721	.329
AFP (>400)	1.750	0.483	6.342	.394
Portal vein thrombosis	0.688	0.213	2.221	.531
Multiple	1.528	0.511	4.567	.448
Comorbidities	3	3	3	3
DM	3.556	0.592	21.358	.166
HT	2.000	0.654	6.113	.224
CAD	1.667	0.370	7.500	.506
CRF	0.467	0.085	2.555	.380
CPC	1.600	0.208	12.281	.651
Cirrhosis	0.964	0.239	3.898	.959
Albumin	2.672	0.756	9.440	.127

*P*: logistic regression; the value in bold signifies significantly higher rate (*P* < .05).

AFP, alpha-fetoprotein; BCLC, Barcelona Clinic of Liver Cancer; CAD, coronary artery disease; CPC, Child–Pugh classification; CRF, chronic renal failure; DM, diabetes mellitus; ECOG, Eastern Cooperative Oncology Group; HT, hypertension.
